# Analysis of the representation of skin tone diversity amongst medical resources illustrating dermatological manifestations of dermatomyositis, systemic sclerosis and vasculitis

**DOI:** 10.1093/rap/rkaf114

**Published:** 2025-10-06

**Authors:** Stacey Kihumba, Giles Dixon, Matthew Wells, Sarah Rudd, Jo Hardy, Harsha Gunawardena, John D Pauling, Huzaifa Adamali, Shaney L Barratt

**Affiliations:** Bristol Medical School, University of Bristol, Bristol, UK; Bristol Interstitial Lung Disease (BILD) Service, North Bristol NHS Trust, Bristol, UK; Department of Clinical & Biomedical Sciences, University of Exeter, Exeter, UK; Bristol Interstitial Lung Disease (BILD) Service, North Bristol NHS Trust, Bristol, UK; Library and Knowledge Service, North Bristol NHS Trust, Bristol, UK; Bristol Interstitial Lung Disease (BILD) Service, North Bristol NHS Trust, Bristol, UK; Bristol Interstitial Lung Disease (BILD) Service, North Bristol NHS Trust, Bristol, UK; Bristol Interstitial Lung Disease (BILD) Service, North Bristol NHS Trust, Bristol, UK; Bristol Interstitial Lung Disease (BILD) Service, North Bristol NHS Trust, Bristol, UK; Bristol Interstitial Lung Disease (BILD) Service, North Bristol NHS Trust, Bristol, UK; Academic Respiratory Unit, Bristol Medical School, University of Bristol, Bristol, UK

**Keywords:** SARD-ILD, DM, SSc, vasculitis, diversity, representation disparities, cutaneous manifestations

## Abstract

**Objectives:**

Cutaneous manifestations of systemic autoimmune rheumatic disease–interstitial lung disease (SARD-ILD) are clinically useful diagnostic features that can support early diagnosis and management. However, medical education resources often lack diversity in representing skin tones, which lead to inequities in healthcare delivery. Our study aimed to quantify the proportion of skin tones represented in medical literature that depict cutaneous features relevant to SARD-ILD.

**Methods:**

A structured search of medical resources was conducted with the support of North Bristol NHS Trust Library and Knowledge Service. We systemically reviewed images of cutaneous signs associated with SSc, DM and vasculitis. Images that did not depict these conditions were excluded. Each image was assigned a Monk Skin Tone (MST) Scale score (1–10). A chi-square goodness-of-fit analysis was used to establish whether the distribution of images was equal.

**Results:**

Sixteen e-resources and 26 textbooks were analysed, yielding 790 images: there were 190 depicting SSc, 401 DM and 199 vasculitis. The chi-square indicated a significantly unequal distribution of images across skin tones (*P* < 0.001), a pattern that persisted across all conditions. Furthermore, there was no significant improvement in skin tone representation from 2009 to 2022 (median skin tone category 1–2).

**Conclusion:**

There is persistent underrepresentation of people of global majority in educational resources, despite evidence of higher disease prevalence and severity among individuals with darker skin tones. Increasing the inclusion of diverse skin tones in medical imagery is essential to enhancing diagnostic accuracy, reducing health disparities, and improving clinical outcomes.

## Graphical abstract

**Figure:**
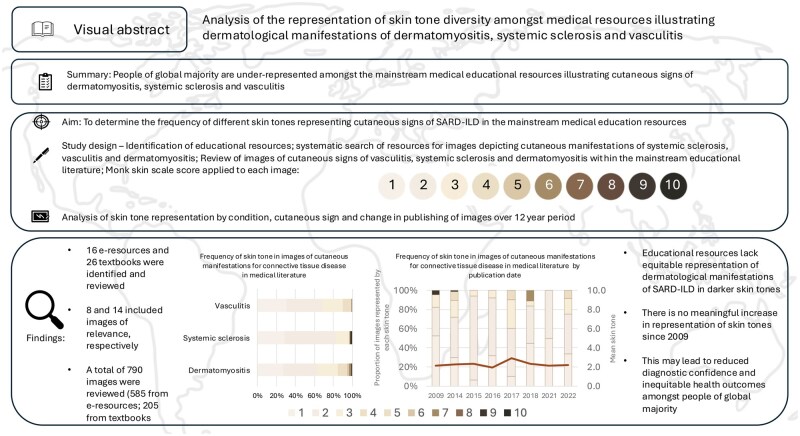

Key messages

- Images used in medical educational resources significantly underrepresent darker skin times, impacting diagnostic accuracy and healthcare equity.
- This includes illustrations of cutaneous signs in SARD despite greater prevalence and greater morbidity amongst those with darker skin tones.
- Despite growing awareness, no significant progress has been made in diversifying dermatological imagery in textbooks over the past decade.

## Introduction

Interstitial lung disease (ILD) encompasses a heterogeneous group of diseases that cause varying degrees of fibrosis and inflammation within the lungs with significant impact upon quality of life. Approximately 30% of ILDs are secondary to a systemic autoimmune rheumatic disease–interstitial lung disease (SARD-ILD) [1]. Recognition of cutaneous manifestations of SARD is essential in differentiating SARD-ILD from other causes of ILD, directly influencing diagnostic pathways, treatment strategies, and prognostic assessments.

Prior research indicates that less than 5% of dermatological images in medical education literature feature darker skin tones [2]. Cutaneous manifestations in individuals with darker skin often present differently, making their underrepresentation problematic [3]. Reduced diagnostic confidence among clinicians and delayed diagnosis in dermatology clinics have been widely reported [4, 5]. Given that people of global majority have a higher incidence and greater morbidity associated with systemic inflammatory disease, failure to accurately depict these manifestations in educational materials may exacerbate existing health disparities [6–9].

This study aimed to quantify and assess representation of diverse skin tones in medical education resources that illustrate SARD-ILD-related cutaneous signs, specifically related to SSc, vasculitis and DM. Additionally, we examined whether any improvements in diversity had been made in educational materials over time.

## Methods

The North Bristol NHS Trust Library and Knowledge Service identified educational resources comprising of subscribed electronic resources, print and e-books included in stock. A systematic search of these resources was performed (searches performed June 2024), and images depicting cutaneous manifestations of SSc, vasculitis and DM were selected (Supplementary Table S1, available at *Rheumatology Advances in Practice* online). Where electronic search was possible, the name of the sign was used in the search; in textbooks, a manual search of the literature was performed.

Monochrome images, poor-quality images or images of signs of relevance observed in patients with alternative aetiology (e.g. petechial rash in meningococcal disease) were excluded from analysis. Each suitable image was assigned a Monk Skin Tone (MST) Scale score [1–10], a validated scale where lower values indicate lighter skin tones [10].

Collected data were analysed by chi-square goodness-of-fit analysis using SPSS version 29 (IBM, USA). The chi-square goodness of fit expected uniform distribution of representation of skin tone. The study was registered with the local quality improvement department (QI10728) but did not require institutional ethics approval.

## Results

A total of 16 e-resources and 26 textbooks were identified, with 8 and 14, respectively, containing relevant images (Supplementary Table S2, available at *Rheumatology Advances in Practice* online). Textbooks were published either in the UK (*n* = 9) or the USA (*n* = 5). Of the 790 images, 585 were from e-resources and 205 from textbooks and were published between 2009 and 2022. Notably, of the 790 images, 401 represented DM, 190 SSc and 199 vasculitis.

The breakdown of the number of images per clinical sign can be seen in Table 1.

**Table 1.** Breakdown of frequency of images for each clinical sign assessed

Clinical sign	Frequency (*n*)
Active Raynaud’s syndrome	58
Calcinosis/calcinosis cutis	38
Digital ulcers/ischaemia	30
Erythema nodosum	12
Gottron’s papules	183
Heliotropic rash	80
Livedo reticularis	25
Mechanic’s hands	33
Microstomia	4
Nailfold capillary dilatation	26
Palmar erythema	20
Palmer papules	5
Periungual overgrowth	12
Puffy fingers	15
Reverse Gottron’s sign	11
Sclerodactyly	23
Shawl/V/Holster sign	48
Splinter haemorrhages	10
Telangiectasia	26
Vasculitic purpuric rash/purpura	82
Vasculitis cutaneous ulceration/nodules	49

### Skin tone representation

**Figure 1.**
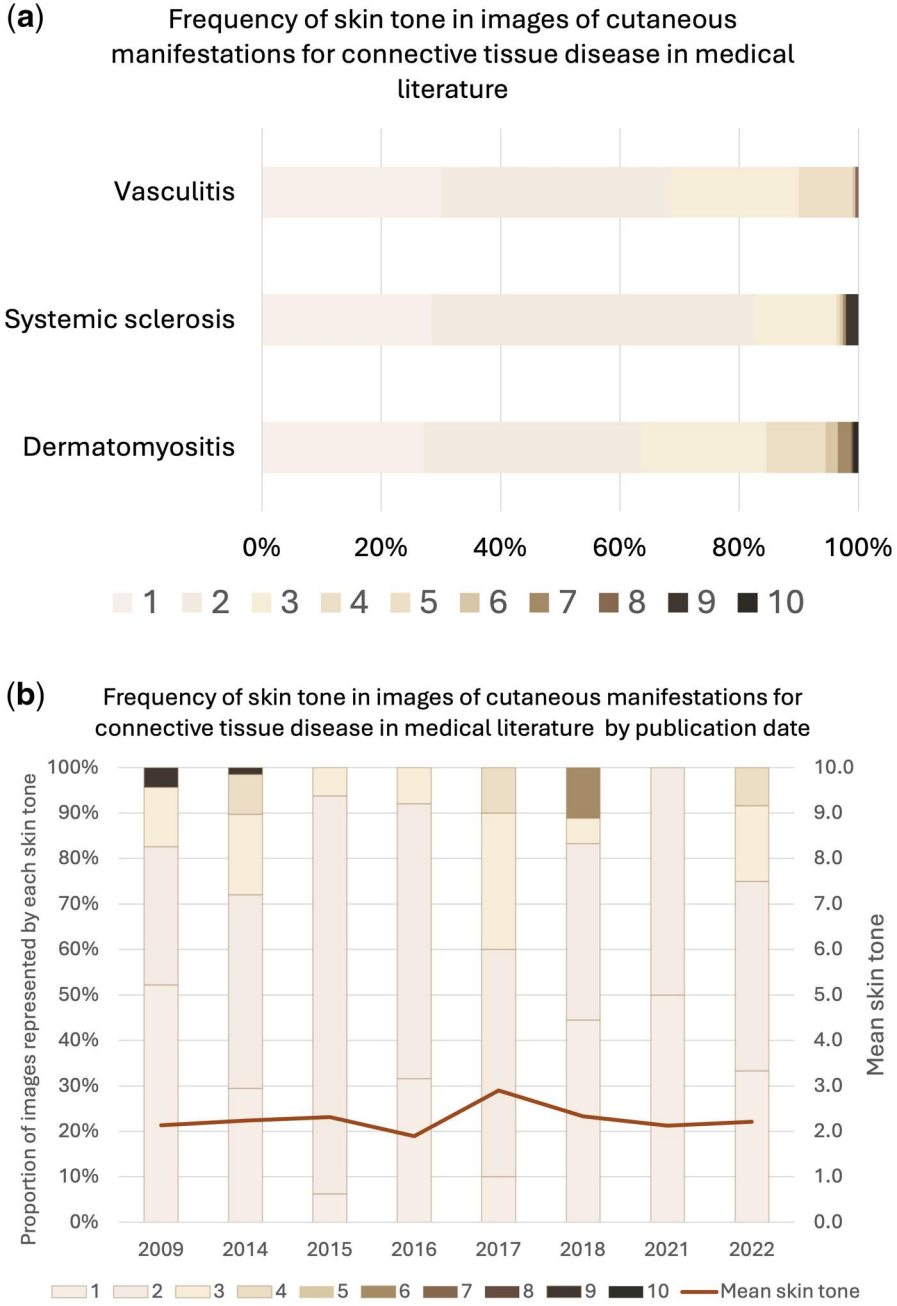
Frequency of skin tone. (**a**) Frequency of skin tone representation in images of cutaneous manifestations for systemic autoimmune rheumatic disease (SARD) in medical literature. Colours represent the percentage of overall image count represented by each Monk Skin Tone. DM (*n* = 401), SSc (*n* = 190) and vasculitis (*n* = 199). (**b**) Frequency of skin tone in images of cutaneous manifestations of connective tissue disease in medical literature by publication date

A significant overrepresentation of lighter skin tones was observed across all clinical signs and disease cohorts (Fig. 1). The chi-square test confirmed a skew towards lighter MST categories, with darker skin tones (higher MST category) largely underrepresented, χ^2^(9) = 1506.8, *P* < 0.001. The standardized residuals identified that tones 1 and 2 were significantly over-represented (16.18 and 27.76, respectively).

### Image representation by publication date

Of the textbooks analysed, the earliest publication year was 2009 and the most recent was 2022. The mean skin tone did not significantly change between 2009 and 2022 (mean MST category 2.3; S.D. ± 0.29).

## Discussion

The study provides compelling evidence that educational resources continue to lack equitable representation of dermatological manifestations of SARD-ILD in darker skin tones, despite extensive literature advocating change since 2006 [11].

Whilst previous work has highlighted this issue, to our knowledge ours is the first study to demonstrate this shortcoming with respect to SARD-ILD. This may be of particular concern given the greater prevalence of many SARDs amongst people of global majority and racial disparities in outcomes [9].

This lack of diversity of skin tones in educational resources is likely to be multifactorial. The presumption that lighter skin demonstrates pathology more clearly may have led to selection bias by authors and publication bias amongst editors. Historically, technical challenges may be contributory; however, modern photographic advancements have allowed for more accurate documentation of cutaneous signs on darker skin tones [12, 13]. The feature of image stabilization in digital cameras has helped to maintain the definition of images capturing dermatological conditions without diminishing colour and degrading lesion margins. Furthermore, utilization of apposite digital camera settings aids in defining colour and limiting interference from the ambient light source [12].

With respect to geographical bias, many medical textbooks originate from regions where lighter skin is predominant, which may influence image selection. The textbooks that contributed the images to the data set were published in the UK and USA, countries in which white individuals account for the largest percentage of their populations: 81.7% and 75.3%, respectively. Despite this, these countries still contain significant populations with darker skin, and patients with darker skin and cutaneous manifestations of SARD-ILD will be present and could be selected for inclusion in relevant literature.

The overrepresentation of lighter skin tones may contribute to diagnostic bias, delayed recognition, and misdiagnosis of cutaneous conditions in patients with darker skin [3, 14]. Research has shown that greater diversity in dermatological education materials improves clinician confidence and diagnostic accuracy, ultimately leading to earlier interventions and better patient outcomes [15].

The need for wider representation of darker skin tones amongst educational resources extends to patients with SARD. Despite awareness of this shortcoming dating back almost 20 years, there appears to have been insufficient progress to enhance representation in medical images over this period [11]. Our study underscores the urgent need for a systemic effort to increase the representation of diverse skin tones in medical education. A concerted effort is necessary to ensure wider representation of skin tones within educational resources, ensure ethical distribution and incorporate these into both the undergraduate and postgraduate medical curriculums. Clinicians and researchers should actively contribute images of dermatological conditions in people of global majority. Importantly, medical organizations should establish guidelines promoting equitable representation in visual medical resources. Future trials should also analyse whether diverse representation in educational resources influences diagnostic accuracy, treatment efficacy and patients’ outcomes, thereby addressing systemic disparities in research.

There are some limitations to the methodology used in the present study. The images were analysed by a single author, introducing the potential for bias. However, previous work has demonstrated a high intraclass correlation coefficient, hopefully minimizing the effect of single review [16]. Future work may consider using the consensus of a panel of clinicians or artificial intelligence to quantify skin tones. However, it is unlikely that subjective variation of skin tone assessment would have significantly altered the findings of this study, which are in keeping with previous findings. Furthermore, no specific sample size calculation was undertaken due to the paucity of pilot or feasibility data in this field.

There are other SARDs which have cutaneous manifestations, such as rheumatoid arthritis and Sjogren’s syndrome. While direct conclusions about these specific conditions cannot be drawn, the pronounced disparity observed in our study suggests that similar patterns are likely to exist in other SARDs.

A relative strength of this project is the structured resource search that incorporated the mainstream educational resources as identified by a large University Hospital library service. Additional work is required to establish whether addressing the shortfall in representation of darker skin tones within the medical literature improves diagnostic performance and outcomes.

This study has highlighted the need for educational resources to be more representative of the populations they serve. This will require significant effort and resources to redress the balance but may contribute to a reduction in healthcare inequality.

## Supplementary Material

rkaf114_Supplementary_Data

## Data Availability

Data available upon request from the corresponding author.
