# Supplementary material for: Analysis of the representation of skin tone diversity amongst medical resources illustrating dermatological manifestations of dermatomyositis, systemic sclerosis and vasculitis
Source: Rheumatol Adv Pract. 2025 Oct 6;9(4):rkaf114. doi: 10.1093/rap/rkaf114 (PMC12531797; doi:10.1093/rap/rkaf114)
Supplement: rkaf114_Supplementary_Data [file rkaf114_supplementary_data.docx]

Supplementary material

Supplementary Table S1 Conditions and the relevant signs searched for within each e-resource or textbook.

| Condition | Sign |
| --- | --- |
| Dermatomyositis | Gottrons’ sign  Heliotropic rash  Holster sign  Mechanic’s hands  Nailfold capillary dilatation  Palmar papules  Periungal overgrowth  Reverse Gottrons’ sign  Shawl sign  V sign |
| Systemic Sclerosis | Active Raynauds’  Calcinosis cutis  Digital ulcers  Digital ischaemia  Microstomia  Nailfold capillary dilatation  Puffy fingers  Sclerodactyly  Telangiectasia |
| Vasculitis | Cutaneous ulceration / nodules  Livedo reticularis  Palmar erythema  splinter haemorrhage  Vasculitic petechia / purpura |

Supplementary Table S2. E-resources and textbooks included containing images of clinical signs highlighted in Supplementary Material S1

| Title | Type | Country of publication | Author | Year of publication |
| --- | --- | --- | --- | --- |
| Ovid MultiMedia search | E-Resource | N/A | N/A | N/A |
| MedPix | E-Resource | N/A | N/A | N/A |
| New England Journal of Medicine Clinical Medicine | E-Resource | N/A | N/A | N/A |
| DynaMed | E-Resource | N/A | N/A | N/A |
| ClinicalKey | E-Resource | N/A | N/A | N/A |
| Wikipedia Commons | E-Resource | N/A | N/A | N/A |
| Images from the History of Medicine (NLM) | E-Resource | N/A | N/A | N/A |
| DermNet | E-Resource | N/A | N/A | N/A |
| Rheumatology / a clinical handbook | Textbook | UK | Al-Sukaini, A | 2014 |
| ABC of rheumatology | Textbook | UK | Adebajo, A | 2018 |
| Rapid review of rheumatology and musculoskeletal disorders | Textbook | UK | Manson, JJ | 2014 |
| Rheumatology (6th edition) | Textbook | UK | Hochberg, MC | 2014 |
| Oxford handbook of rheumatology (4th edition) | Textbook | UK | Clunie, G | 2018 |
| Firestein & Kelley's textbook of rheumatology | Textbook | US | Firestein, GS | 2009 |
| Murray and Nadel's textbook of respiratory medicine (6th edition) | Textbook | US | Courtney Broaddus, V | 2016 |
| Oxford handbook of medical dermatology (2nd edition ) | Textbook | UK | Burge, S | 2016 |
| Clinical dermatology (5th edition ) | Textbook | UK | Weller, RB | 2015 |
| Dermatology : an illustrated colour text (7th edition) | Textbook | UK | Gawkrodger, DJ | 2021 |
| Dermatology made easy | Textbook | UK | Oakley, A | 2017 |
| Dermatology | Textbook | US | Craythorne, E | 2015 |
| Dermatology essentials (2nd edition) | Textbook | US | Bolognia, JL | 2022 |
| Clinical dermatology : a color guide to diagnosis and therapy (6th edition) | Textbook | US | Habif, TP | 2016 |
